# Impaired Kidney Function and 10-Year Outcome After Percutaneous Coronary Intervention—Interaction with Age, Sex, Diabetic Status and Clinical Presentation

**DOI:** 10.3390/jcm13226833

**Published:** 2024-11-13

**Authors:** Gjin Ndrepepa, Sebastian Kufner, Salvatore Cassese, Michael Joner, Hendrik B. Sager, Erion Xhepa, Karl-Ludwig Laugwitz, Heribert Schunkert, Adnan Kastrati

**Affiliations:** 1 Department of Cardiology, Deutsches Herzzentrum München, TUM Universitätsklinikum, Lazarettstrasse 36, 80636 München, Germany; sebastian.kufner@tum.de (S.K.); cassese@dhm.mhn.de (S.C.); joner@dhm.mhn.de (M.J.); hendrik.sager@tum.de (H.B.S.); xhepa@dhm.mhn.de (E.X.); schunkert@dhm.mhn.de (H.S.); kastrati@dhm.mhn.de (A.K.); 2 German Center for Cardiovascular Research (DZHK), Partner Site Munich Heart Alliance, Munich, Germany; laugwitz@mytum.de; 3 Medizinische Klinik und Poliklinik Innere Medizin I (Kardiologie, Angiologie, Pneumologie), Klinikum rechts der Isar, Technische Universität München, Ismaninger Str. 22, 81675 München, Germany

**Keywords:** chronic kidney disease, coronary artery disease, glomerular filtration rate, mortality: percutaneous coronary intervention

## Abstract

**Background**: Limited evidence exists regarding the association of chronic kidney disease (CKD) with long-term outcomes following percutaneous coronary intervention (PCI). We aimed to assess the association of CKD with 10-year outcome after PCI. **Methods:** This study included 5571 patients with coronary artery disease (CAD) undergoing PCI. Patients were categorized in groups according to the estimated glomerular filtration rate (eGFR) values: eGFR ≥ 90 mL/min/1.73 m^2^, (normal kidney function), 60 to <90 mL/min/1.73 m^2^ (mild kidney impairment), 30 to <60 mL/min/1.73 m^2^ (mild-to-moderate and moderate-to-severe kidney impairment) and <30 mL/min/1.73 m^2^ (severe kidney impairment). The primary endpoint was all-cause mortality at 10 years. **Results:** All-cause deaths occurred in 155 patients (86.3%) with eGFR < 30 mL/min/1.73 m^2^, 602 patients (59.1%) with eGFR 30 to <60 mL/min/1.73 m^2^, 775 patients (31.3%) with eGFR 60 to <90 mL/min/1.73 m^2^ and 220 patients (15.8%) with eGFR ≥ 90 mL/min/1.73 m^2^ (adjusted hazard ratio = 2.16, 95% confidence interval 1.84 to 2.54, *p* < 0.001, for 30 mL/min/1.73 m^2^ decrement in the eGFR). There were CKD-by-age (Pint < 0.001) and CKD–by-clinical presentation (Pint = 0.017) interactions showing a stronger association of CKD with mortality in younger patients and those presenting with acute coronary syndromes. The C statistic of the multivariable model for mortality increased from 0.748 [0.737–0.759] to 0.766 [0.755–0.777] (*p* < 0.001) after the inclusion of eGFR in the model. **Conclusions:** In patients with CAD undergoing PCI, CKD was associated with higher mortality at 10 years compared with patients with preserved renal function. The association between CKD and mortality was stronger in patients of younger age and those presenting with acute coronary syndromes.

## 1. Introduction

Chronic kidney disease (CKD) affects approximately 700 million people worldwide, and its prevalence is projected to increase due to demographic trends (population growth and aging), increased burden of main drivers of CKD (diabetes mellitus, arterial hypertension, obesity and cardiovascular disease) and climate change [1]. CKD is known to increase the risk of mortality and other adverse outcomes in patients with cardiovascular disease [2,3] and cardiovascular disease is a leading cause of mortality in patients with CKD [4]. The prevalence of coronary artery disease (CAD) is nearly 2-fold higher in individuals with CKD compared with individuals without CKD [5]. In the past, the association of CKD with the outcome of patients with CAD has been extensively investigated, yet a number of issues still remain. Historically, CKD patients have been underestimated in cardiovascular clinical studies and the representation of patients with CKD in randomized cardiovascular trials has not improved in the past 2 decades [6]. Patients with CAD and CKD receive guideline-recommended therapies including revascularization by percutaneous coronary intervention (PCI) [7,8] less often due to fears of contrast-induced acute kidney injury, although they appear to benefit from these therapies [9,10]. Patients with CKD receive potent antithrombotic drugs less often due to fear of bleeding and they are more likely to discontinue dual antiplatelet therapy in the first year after PCI compared with patients without CKD [11]. CKD has mostly been defined as a glomerular filtration rate (GFR) of less than 60 mL/min [11,12,13] and the association between mild reduction of renal function (GFR between 60 and <90 mL/min) and outcome after coronary revascularization is less often investigated and data are controversial [14,15]. Of note, the highest GFR value associated with an increase in the risk of mortality after PCI is unknown. The association between CKD and mortality after PCI has been investigated in studies with a short-term follow-up and few studies have had a follow-up longer than 5 years [16,17,18]. While studies are concordant with respect to the association between CKD and mortality after PCI, the association of CKD with other outcomes like myocardial infarction, stent thrombosis or need for revascularization remains poorly investigated. Finally, whether the association between CKD and outcome after PCI differs according to age, sex, diabetic status and clinical presentation (acute coronary syndrome [ACS] or chronic coronary disease [CCD]) is unknown. In this study, we aimed first to assess the association of renal function with 10-year clinical outcome after PCI across the whole spectrum of GFR, and second, to investigate whether there are CKD-by-age, -sex, -diabetic status or -clinical presentation interactions in terms of association with 10-year outcome after PCI.

## 2. Methods

### 2.1. Patients

This retrospective study included 5571 patients with CAD treated with stent implantation from September 2007 to August 2009 in 2 tertiary hospitals in Munich, Germany. Patients were recruited in 2 randomized studies conducted to assess the efficacy of drug-eluting stents: the Intracoronary Stenting and Angiographic Results: Test Efficacy of 3 Limus-Eluting Stents (ISAR-TEST 4 trial; NCT00598676; 2603 patients) [19] and the Intracoronary Stenting and Angiographic Results: Test Efficacy of Sirolimus- and Probucol- and Zotarolimus-Eluting Stents (ISAR-TEST 5 trial; NCT00598533; 3002 patients) [20]. These trials enrolled patients ≥18 years of age who presented with ischemic symptoms or had evidence of spontaneous or inducible myocardial ischemia. All included patients had angiographic documentation of significant CAD (stenoses with ≥50% lumen obstruction located in the native coronary arteries). Patients with a target lesion located in the left main coronary artery, cardiogenic shock, cancer (or other concomitant diseases) with a life expectancy of less than 1 year, documented allergy to the study drugs or pregnancy were excluded. Of the 5605 patients fulfilling these inclusion/exclusion criteria, 5571 patients had estimated GFR (eGFR) data available and these patients are included in the current study. PCI was performed via the femoral artery approach in all patients. Written informed consent and institutional ethics committee approval were obtained in the setting of primary studies [19,20]. This study conforms to the Declaration of Helsinki. The data that support the findings of this study are available from the corresponding author upon reasonable request.

### 2.2. Definitions and Measurements

CAD was diagnosed by angiographic documentation of coronary artery narrowings with ≥50% lumen obstruction in the native coronary arteries in the presence of symptoms or documentation of spontaneous or inducible myocardial ischemia. Arterial hypertension, hyperlipidemia, type 2 diabetes and current smoking were defined according to the respective guidelines at the time of patient’s enrollment in the primary studies. Body mass index was calculated using the patient’s height and weight measured during the hospital course. The global left ventricular ejection fraction was measured by left ventricular angiography using the area-length method. Serum creatinine was measured before coronary angiography using a kinetic colorimetric assay according to the compensated Jaffe method. GFR was calculated using the Chronic Kidney Disease Epidemiology Collaboration equation (CKD-EPI) [21]. Patients were categorized according to the eGFR values: eGFR ≥ 90 mL/min/1.73 m^2^ (normal kidney function), 60 to <90 mL/min/1.73 m^2^ (mild kidney impairment), 30 to <60 mL/min/1.73 m^2^ (mild-to-moderate and moderate-to-severe kidney impairment) and <30 mL/min/1.73 m^2^ (severe kidney impairment).

### 2.3. Outcomes and Follow-Up

The primary endpoint was mortality at 10 years. Cardiac mortality, noncardiac mortality, myocardial infarction, definite stent thrombosis, target lesion revascularization (TLR), target vessel revascularization (TVR) and nontarget vessel revascularization (NonTVR) at 10 years were also analyzed. Cardiac death and definite stent thrombosis were defined according to the Academic Research Consortium (ARC) criteria [22]. All other deaths were classified as noncardiac deaths. Myocardial infarction was defined according to the 2007 Universal Definition of Myocardial Infarction criteria [23]. TLR was defined as repeat balloon angioplasty or stent implantation in a stented lesion including 5 mm borders adjacent to the stent. TVR was defined as a repeat PCI or bypass surgery of any segment of the target vessel, including the target lesion. NonTVR was defined as a repeat revascularization of a coronary vessel other than the target vessel.

The follow-up included telephone calls or office visits at 1 month and 1 year after index PCI and annually up to 10 years. Events at follow-up were adjudicated and classified by an event adjudication committee blinded to patients’ data in the setting of primary trials.

### 2.4. Statistical Analysis

Continuous data are shown as median with 25th–75th percentiles after the assessment of normality of distribution by the Kolmogorov–Smirnov test. Continuous data with skewed distribution were compared using the Kruskal–Wallis rank sum test. Categorical data are shown as counts and proportions (%) and compared with the chi-squared test. Deaths at 10 years are shown as cumulative incidences. Other outcomes are shown as cumulative incidences after considering the impact of competing risk of death. The univariable Cox proportional hazards model was used to compare outcomes in groups according to the eGFR values. The association of eGFR with the outcomes was assessed using the multivariable Cox proportional hazards model. All variables that differed significantly between the groups in Table 1 and Supplementary Table S1 plus hypercholesterolemia were entered into the multivariable Cox proportional hazards model. eGFR was entered into the Cox proportional hazards model as a continuous variable. Missing values of baseline variables were imputed using predictive mean matching. Eventual differences in the association of CKD (defined as an eGFR of <60 mL/min/1.73 m^2^) and outcomes in patients’ subgroups according to age (< 75 years vs. ≥75 years), sex (women vs. men), diabetes mellitus (yes vs. no) and clinical presentation (ACS vs. no ACS) were investigated by performing interaction testing. A nonlinear relationship between eGFR and mortality was assessed by performing the unadjusted and adjusted restricted cubic spine regression analysis with 30, 60 and 90 mL/min/1.73 m^2^ eGFR knots. Computed *p* for nonlinearity < 0.05 was considered to indicate a nonlinear relationship. The discrimination with respect to mortality added by eGFR inclusion into the multivariable model alongside relevant clinical and epidemiological variables was assessed by calculating and comparing the C statistics of the multivariable Cox proportional hazards models applied for mortality without (with baseline variables only) and with inclusion of eGFR (baseline variables plus eGFR). The C statistics of the models with and without eGFR were compared using the CompareC package. All statistical analyses were performed using the R 4.1.0 Statistical Software (The R Foundation for Statistical Computing, Vienna, Austria). A two-sided *p* < 0.05 was considered statistically significant.

## 3. Results

### 3.1. Baseline Data

This study included 5571 patients. Patients were categorized according to the following eGFR cut-offs: a group with eGFR <30 mL/min/1.73 m^2^ (198 patients), a group with eGFR 30 to <60 mL/min/1.73 m^2^ (1104 patients), a group with eGFR 60 to <90 mL/min/1.73 m^2^ (2684 patients) and a group with eGFR ≥90 mL/min/1.73 m^2^ (1585 patients). Baseline variables are shown in Table 1. All baseline variables with the exception of hypercholesterolemia, oral antidiabetic drugs and proportion of diabetic patients on diet alone appear to differ significantly in groups according to eGFR cut-offs. Procedural data are shown in Table S1. There appear to be significant differences between the groups with respect to the treated coronary vessel and lesion complexity. The remaining characteristics appear to differ little across the groups. After PCI, patients were prescribed 200 mg/day aspirin indefinitely and a thienopyridine (predominantly clopidogrel 150 mg/day for the first 3 days, followed by 75 mg/day for at least 6 months). Other drugs were prescribed at the discretion of the treating physician (Table S2). Aspirin, P2Y_12_ inhibitors, statins, angiotensin-converting enzyme inhibitors and beta blocking agents were prescribed less often in patients with moderate-to-severe (eGFR: 30 to <60 mL/min/1.73 m^2^) and severe (<30 mL/min/1.73 m^2^) impairment of renal function compared with patients with mild impairment or preserved renal function.

### 3.2. Clinical Outcome

**Figure 1:**
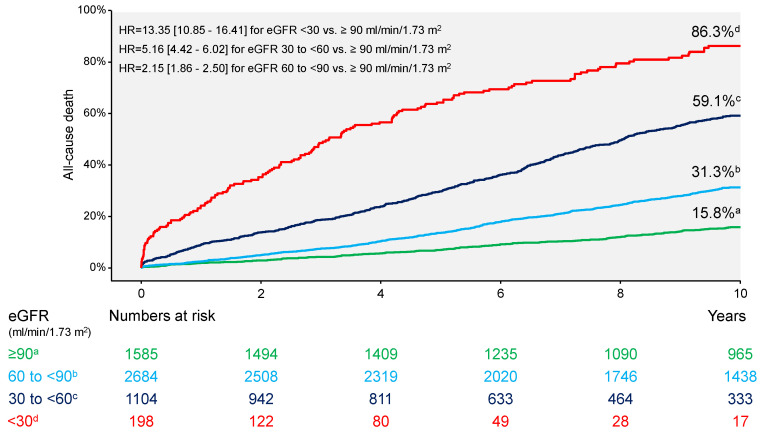
Ten-year incidence of all-cause mortality. eGFR = estimated glomerular filtration rate; HR = hazard ratio.

**Figure 2:**
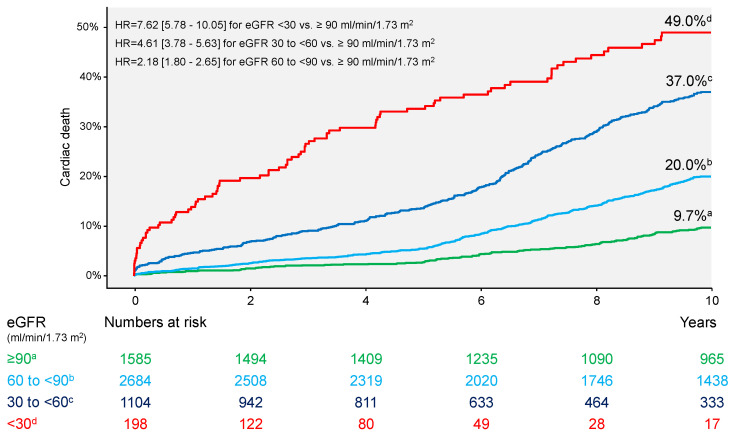
Ten-year incidence of cardiac mortality. eGFR = estimated glomerular filtration rate; HR = hazard ratio.

Clinical events at 10 years are shown in Table 2. Overall, there were 1752 deaths (31.4%). Patients who died had a significantly lower eGFR compared with patients who were alive at 10 years (64.5 [46.8–81.8] vs. 84.1 [68.7–94.3] ml/min/1.73 m^2^; *p* < 0.001). All-cause deaths occurred in 155 patients (86.3%) with eGFR <30 mL/min/1.73 m^2^, 602 patients (59.1%) with eGFR 30 to <60 mL/min/1.73 m^2^, 775 patients (31.3%) with eGFR 60 to <90 mL/min/1.73 m^2^ and 220 patients (15.8%) with eGFR ≥90 mL/min/1.73 m^2^ (overall *p* value <0.001; Figure 1). Cardiac deaths occurred in 1073 patients: 87 patients (49.0%) with eGFR <30 mL/min/1.73 m^2^, 369 patients (37.0%) with eGFR 30 to <60 mL/min/1.73 m^2^, 486 patients (20.0%) with eGFR 60 to <90 mL/min/1.73 m^2^ and 131 patients (9.7%) with eGFR ≥90 mL/min/1.73 m^2^ (overall *p* value <0.001; Figure 2). Noncardiac deaths occurred in 679 patients (39% of deaths): 68 patients (37.3%) with eGFR <30 mL/min/1.73 m^2^, 233 patients (22.1%) with eGFR 30 to <60 mL/min/1.73 m^2^, 289 patients (11.3%) with eGFR 60 to <90 mL/min/1.73 m^2^ and 89 patients (6.1%) with eGFR ≥90 mL/min/1.73 m^2^ (overall *p* value < 0.001; Figure S1). The incidence of myocardial infarction appears to increase with deterioration of renal function (overall *p* value = 0.002; Table 2 and Figure S2). The remaining outcomes appear to differ little in groups according to eGFR values (Table 2 and Figures S3 to S6). Patients with an eGFR of <15 mL/min/1.73 m^2^ (*n* = 75) had the highest risk of death at 10 years (65 deaths; Kaplan–Meier estimate of 10-year mortality, 92.4%).

**Figure 3:**
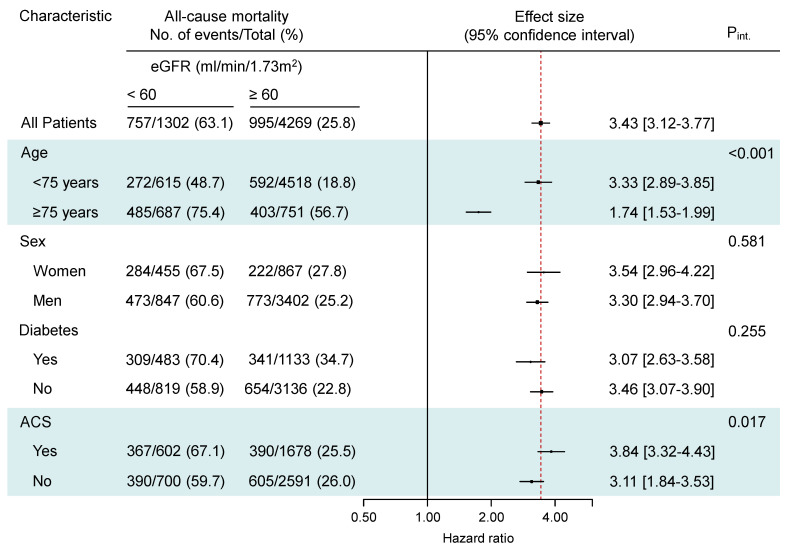
All-cause mortality in subgroups of patients according to age, sex, diabetic status and clinical presentation. ACS = acute coronary syndrome; P_int_ = P for interaction. The highlights show significant interactions.

**Figure 4:**
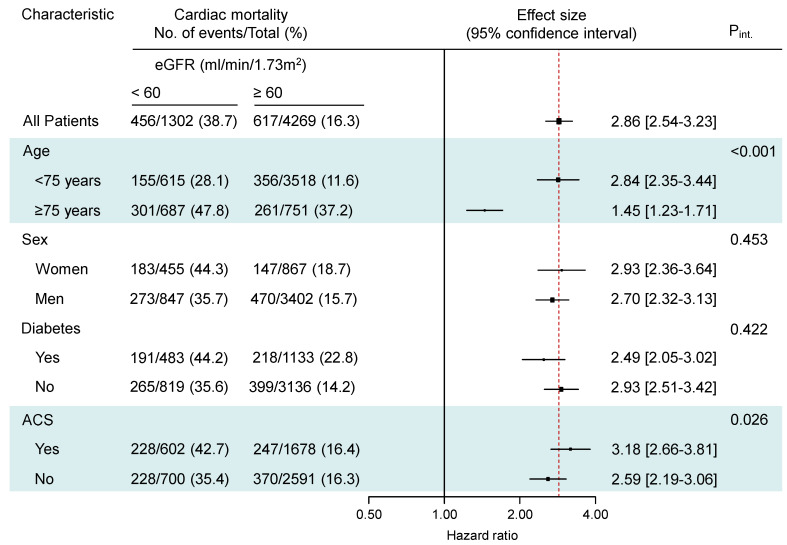
Cardiac mortality in subgroups of patients according to age, sex, diabetic status and clinical presentation. ACS = acute coronary syndrome; P_int_ = P for interaction. The highlights show significant interactions.

There were eGFR-by-age and eGFR-by-clinical presentation interactions showing a stronger association of reduced eGFR with the risk for all-cause and cardiac mortality in patients younger than 75 years (P_int_ < 0.001 for both all-cause and cardiac mortality) and patients presenting with an ACS (P_int_ = 0.017 for all-cause mortality and P_int_ = 0.026 for cardiac mortality; Figure 3 and Figure 4). The association of reduced eGFR with the risk of noncardiac mortality and myocardial infarction was also stronger in patients < 75 years of age (P_int_ < 0.001 for noncardiac mortality and P_int_ = 0.022 for myocardial infarction; Figures S7 and S8). The association of reduced eGFR with the risk of stent thrombosis was stronger in women with reduced eGFR compared with men (P_int_ = 0.012; Figure S9). There were no significant interactions between eGFR and age, sex, diabetic status or clinical presentation with respect to the risk for TLR (P_int_ > 0.547 for all interactions), TVR (P_int_ > 0.371 for all interactions) and NonTVR (P_int_ > 0.100 for all interactions).

The eGFR–mortality association was adjusted in the multivariable Cox proportional hazards model (see Methods for variables we adjusted for). eGFR was associated with the risk for all-cause mortality (adjusted hazard ratio [HR] = 2.16, 95% confidence interval 1.84 to 2.54, *p* < 0.001), cardiac mortality (adjusted HR = 2.15 [1.75–2.64], *p* < 0.001) and noncardiac mortality (adjusted HR = 2.21 [1.71–2.86], *p* < 0.001). Full results of Cox proportional hazards model are shown in Table S3. The association between eGFR and mortality remained significant after the inclusion in the multivariable model of main cardiovascular drugs at hospital discharge (aspirin, P2Y_12_ inhibitors, angiotensin-converting enzyme inhibitors, angiotensin II receptor blockers, beta-blocking agents and diuretic drugs) with the following adjusted HR for all-cause (adjusted HR = 1.65 [1.50–1.81]), cardiac (adjusted HR = 1.62 [1.43–1.83]) and noncardiac (adjusted HR = 1.73 [1.49–2.00]) mortality; all risk estimates were calculated for 30 mL/min decline in the eGFR. The C statistics of the multivariable model with baseline variables (without eGFR) applied for 10-year all-cause, cardiac and noncardiac mortality were 0.748 [0.737–0.759], 0.767 [0.753–0.781] and 0.728 [0.710–0.746], respectively. The inclusion of eGFR in the model alongside baseline variables led to a significant increase of the C statistics for all-cause (C statistic: 0.766 [0.755–0.777], *p* < 0.001), cardiac (C statistic: 0.783 [0.770–0.796], *p* < 0.001) and noncardiac mortality (C statistic: 0.747 [0.730–0.765], *p* < 0.001). The association of impaired renal function eGFR categories with clinical outcome (as compared with the preserved renal function category) is shown in Table S4.

**Figure 5:**
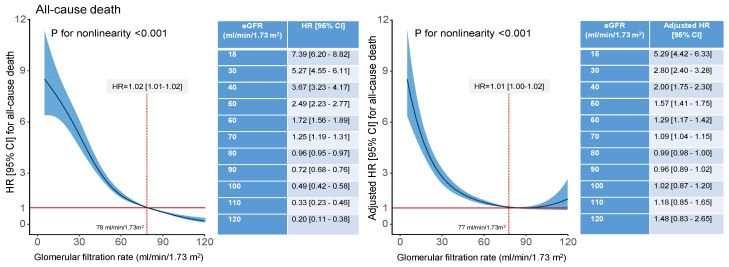
Unadjusted (**left** panel) and adjusted (**right** panel; the list of variables is shown in the Methods) association between glomerular filtration rate (GFR) and all-cause mortality. The spline curves show the association between GFR values up to 120 mL/min/1.73 m^2^ and all-cause mortality at 10 years. The inserted tables on the right side of each graph show unadjusted (left panel) and adjusted (right panel) hazard ratios for all-cause mortality for GFR values between 15 and 120 mL/min/1.73 m^2^. For GFR values lower than 78 mL/min/1.73 m^2^ (in unadjusted analysis) and 77 mL/min/1.73 m^2^ (in adjusted analysis), the association between GFR and 10-year all-cause mortality was significant. CI = confidence interval; eGFR = estimated glomerular filtration rate; HR = hazard ratio.

**Figure 6:**
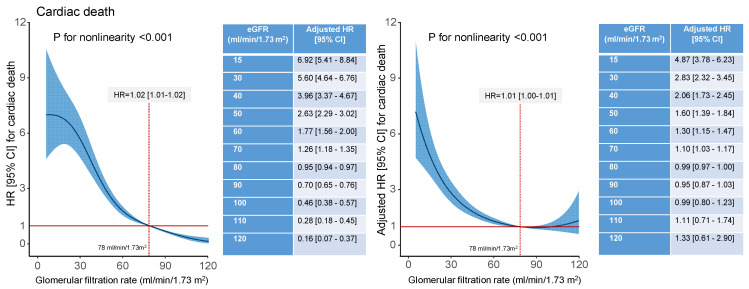
Unadjusted (**left** panel) and adjusted (**right** panel; the list of variables is shown in the Methods) association between glomerular filtration rate (GFR) and cardiac mortality. The spline curves show the association between GFR values up to 120 mL/min/1.73 m^2^ and cardiac mortality at 10 years. The inserted tables on the right side of each graph show unadjusted (left panel) and adjusted (right panel) hazard ratios for cardiac mortality for GFR values between 15 and 120 mL/min/1.73 m^2^. For GFR values lower than 78 mL/min/1.73 m^2^ the association between GFR and 10-year cardiac mortality was significant in unadjusted and adjusted analysis. CI = confidence interval; eGFR = estimated glomerular filtration rate; HR = hazard ratio.

The unadjusted and adjusted restricted cubic spline regression analysis showed a nonlinear association between eGFR and all-cause, cardiac and noncardiac mortality (*p* for nonlinearity < 0.001 for all three relationships). In unadjusted analysis, for eGFR values <78 mL/min/1.73 m^2^, the association of eGFR and all-cause, cardiac and noncardiac mortality was significant. In adjusted analysis, eGFR values <77 mL/min/1.73 m^2^, <78 mL/min/1.73 m^2^ and <70 mL/min/1.73 m^2^ were significantly associated with higher risk of all-cause, cardiac and noncardiac mortality, respectively (Figure 5, Figure 6 and Figure S10).

## 4. Discussion

The principal findings of the current study can be summarized as follows: (1) in patients with CAD undergoing PCI, impaired renal function was associated with a higher risk of all-cause, cardiac and noncardiac mortality at 10 years. The adjusted risk for all-cause, cardiac and noncardiac mortality appears to increase by more than 2-fold for every 30 mL/min/1.73 m^2^ reduction in the GFR. The eGFR values lower than 78 mL/min/1.73 m^2^ were associated with a higher risk of all-cause and cardiac mortality whereas eGFR values lower than 70 mL/min/1.73 m^2^ were associated with a higher adjusted risk of noncardiac mortality. (2) There was an eGFR-by-age and eGFR-by-clinical presentation interaction suggesting a stronger association of reduced GFR with the risk for all-cause and cardiac mortality in patients of younger age and those presenting with an ACS. The interaction testing also showed that the association of reduced GFR with the risk of noncardiac mortality or myocardial infarction was stronger in patients of younger age. There was no CKD-by-sex or CKD-by-diabetic status interaction regarding mortality suggesting a similar strength of association between CKD and mortality in both sexes and patients with and without diabetes. (3) There was no significant association between reduced GFR and 10-year incidence of TLR, TVR or NonTVR.

Previous studies offered strong evidence regarding the association between CKD and the increased risk of mortality after PCI in various clinical scenarios including patients with CCD, ACS, chronic total occlusion, acute myocardial infarction, diabetes and elderly with ACS [24,25,26,27,28,29,30]. These studies assessed the association of CKD with the risk of mortality after coronary revascularization at a short-term follow-up (from inhospital up to 3 years). Since patients with CAD and CKD have more cardiovascular risk factors and comorbidities than patients with preserved kidney function [24], it is plausible that the association of CKD with mortality may be attenuated over time due to the competing effect of cardiovascular risk factors and comorbidities (that tend to be more frequent in patients with CKD). Two small studies with 371 patients with CAD undergoing PCI [16] and 375 patients with ACS undergoing PCI or coronary artery bypass surgery [18] showed that patients with CKD had a higher risk for all-cause and cardiac mortality compared with patients with preserved renal function over a 9-year [16] and 10-year [18] follow-up, respectively. Along the same lines, a prospective registry of all-comers undergoing PCI showed a strong correlation between CKD and mortality or major adverse cardiovascular events over a 7-year follow-up [17]. Apart from corroborating the findings of these studies, our study offers evidence that the risk for mortality conferred by CKD persists up to 10 years after revascularization by PCI. The higher risk for mortality in patients with CKD may be explained by increased cardiometabolic risk that goes in parallel with the severity of CKD [31]. Putative mechanisms of the increased cardiometabolic risk and mortality in patients with impaired renal function are shown in Supplemental Figure S11.

With regard to the association between CKD and long-term mortality, several findings of our study deserve comment. First, time-to-event curves of mortality expand over time showing that the differences in mortality in groups according to eGFR value increase over time at least up to 10 years. Second, although a GFR value of <60 mL/min/1.73 m^2^ has been widely used to define CKD, our study showed that in patients with CAD treated with PCI, the risk of mortality appears to increase for GFR values much higher than this GFR cut-off. This finding is supported by a recent study in patients with CAD and mild-to-moderate CKD treated with PCI, which showed a graded increase in the risk of mortality for GFR values <75 mL/min/1.73 m^2^ [32]. Similar findings were also reported in patients after myocardial infarction [2] and the general population [33]. Third, the inclusion of GFR in the models of mortality increased discrimination by these models with respect to prediction of all-cause, cardiac and noncardiac mortality. Thus, in CAD patients undergoing PCI, reduced GFR provides prognostic information that is independent of and beyond the prognostic information offered by traditional cardiovascular risk factors and other relevant epidemiological and clinical variables. Fourth, interaction testing revealed that the association of impaired renal function with mortality was stronger in patients of younger (<75 years) age and those presenting with an ACS. A number of previous studies have shown an attenuation of the risk of death associated with CKD with advancing age [34,35,36]. A greater risk of inhospital [37] and one-year [38] mortality associated with renal insufficiency in younger patients with acute myocardial infarction has also been demonstrated. Another recent study has reported a CKD-by-age interaction showing reduced likelihood of receiving revascularization in older patients with CKD [39]. Despite this evidence, the mechanisms of a stronger association of CKD with mortality in younger patients are unknown. A greater impact of risk factors like type 2 diabetes and arterial hypertension in patients of younger age compared with older patients and the possibility that the risk of death due to CKD in the elderly is likely diluted by more prevalent comorbidities have been suggested [37]. We also hypothesize that a reduced GFR in patients of younger age may be due to the renal disease per se which may be more malign in terms of association with mortality than age-related decline in the renal function observed in the elderly patients. However, this hypothesis needs testing. To our knowledge, the GFR-by-clinical presentation interaction showing a stronger association between reduced GFR and mortality in patients presenting with an ACS than CCD has not been reported before. Although the underlying mechanisms for this interaction are unknown, it has been suggested that patients with CKD are more likely to present with an acute rather than a stable form of CAD [40] and that patients with ACS have a predisposition to recurrent coronary events [41]. Thus there may be a synergism between CKD and presentation with ACS, with both conditions increasing the risk of subsequent coronary events and mortality.

Our study assessed other outcomes after PCI. We found a graded increase in the 10-year incidence of myocardial infarction with decreasing GFR values and a higher risk of stent thrombosis particularly in patients with severe CKD. These findings appear to reflect increased thrombotic risk in patients with CKD [42]. Patients with CAD and CKD are characterized by an earlier onset and more rapid progression of atherosclerosis [43] and their coronary atherosclerotic plaques have a greater necrotic core and less fibrous tissue compared with patients without CKD [44,45]. However, our study did not find a significant difference in the rates of ischemia-driven repeat revascularizations in patients with various GFR values. Our findings are supported by previous studies that have reported no difference in TVR, restenosis or adverse events related to nonculprit lesions in patients with CKD versus patients without CKD [15,24,45]. In fact, we found numerically lower rates of TLR, TVR and NonTVR with decreasing GFR values. Although the reasons for not finding an association between reduced GFR and the need for repeat revascularization after PCI are unknown, two suggestions may be made. First, reluctance to perform PCI in patients with CKD even in the presence of indication due to fears of contrast-induced acute kidney injury and bleeding. Second, an obscuring effect of mortality, i.e., patients who died might have had more neointimal hyperplasia or progression of atherosclerosis requiring intervention. However, these events may remain undocumented in dying patients.

A number of limitations should be mentioned. First, although the study patients were obtained from two randomized clinical trials with rigorous criteria for follow-up and event adjudication, this study has a retrospective design. Second, all included patients underwent a single management strategy (i.e., PCI). Thus, differences in the efficacy of various management strategies in patients with CAD and CKD cannot be assessed. Third, conditional on the inclusion criteria and outcomes of the primary studies, CKD progression, the use of dialysis and outcomes such as bleeding or stroke cannot be assessed in the setting of current study. Fourth, we had no data available with respect to the volume or type of contrast media used during the PCI procedures and the incidence of acute kidney injury following PCI. Fifth, since the operators’ names were not recorded in the electronic case report forms (eCRFs), the impact of operator’s experience on the clinical outcome [46] after PCI cannot be assessed. Sixth, sodium-glucose contransporter 2 inhibitor (SGLT-2) drugs were not available at the time of patients’ recruitment for the source studies. As a consequence, the cardiac and renal benefits of these drugs in CAD patients with and without diabetes mellitus [47] cannot be assessed. Seventh, although we adjusted for all available epidemiological and clinical data, an eventual impact of residual confounding on the association of CKD with mortality cannot be neglected. Although not desirable, in our view, these limitations do not interfere with the principal findings of this study.

In conclusion, in CAD patients treated with PCI, CKD was associated with an increased risk of all-cause, cardiac and noncardiac mortality at 10 years after the coronary intervention. The association of impaired renal function with mortality was stronger in patients of younger age and patients presenting with an ACS. The need for ischemia-driven repeat revascularizations in treated and untreated coronary arteries appear not to differ according to the CKD status.

## Figures and Tables

**Table 1:** Baseline characteristics.

Characteristic	Estimated Glomerular Filtration Rate (mL/min/1.73 m^2^)				*p* Value
	<30 (*n* = 198)	30 to <60 (*n* = 1104)	60 to <90 (*n* = 2684)	≥90 (*n* = 1585)	
Estimated GFR (mL/min/1.73 m^2^)	21.0 [10.1–26.7]	50.1 [42.8–55.2]	76.9 [68.8–84.5]	96.9 [93.4–102]	<0.001
Age (years)	75.3 [68.1–81.0]	75.5 [69.3–81.4]	69.8 [64.3–75.5]	59.2 [52.5–64.6]	<0.001
Women	68 (34.3%)	387 (35.1%)	630 (23.5%)	237 (15.0%)	<0.001
History of arterial hypertension	127 (64.1%)	792 (71.7%)	1877 (69.9%)	965 (60.9%)	<0.001
History of hypercholesterolemia	118 (59.6%)	713 (64.6%)	1735 (64.6%)	1037 (65.4%)	0.452
Diabetes mellitus	102 (51.5%)	381 (34.5%)	692 (25.8%)	441 (27.8%)	<0.001
On insulin therapy	70 (35.4%)	165 (14.9%)	193 (7.2%)	106 (6.7%)	<0.001
On oral antidiabetic drugs	22 (11.1%)	165 (14.9%)	377 (14.0%)	256 (16.2%)	0.130
On diet alone	10 (5.0)	51 (4.6)	122 (4.6)	79 (5.0)	0.919
Body mass index (kg/m^2^)	26.2 [23.8–29.7]	26.9 [24.5–29.9]	26.9 [24.5–29.8]	27.4 [24.8–30.5]	<0.001
Current smoker	15 (7.6%)	95 (8.6%)	362 (13.5%)	459 (29.0%)	<0.001
Prior myocardial infarction	70 (35.4%)	355 (32.2%)	768 (28.6%)	430 (27.1%)	0.007
Prior coronary artery bypass surgery	16 (8.1%)	172 (15.6%)	244 (9.1%)	109 (6.9%)	<0.001
Diagnosis at presentation					<0.001
Chronic coronary disease	99 (50.0%)	601 (54.4%)	1655 (61.7%)	936 (59.1%)	
Acute coronary syndrome	99 (50.0%)	503 (45.6%)	1029 (38.3%)	649 (40.9%)	
Serum creatinine (mg/dl)	2.63 [2.12–4.81]	1.30 [1.20–1.50]	1.00 [0.85–1.07]	0.80 [0.70–0.89]	<0.001
Number of coronary arteries narrowed 1 2 3	21 (10.6%) 36 (18.2%) 141 (71.2%)	121 (11.0%) 252 (22.8%) 731 (66.2%)	418 (15.6%) 719 (26.8%) 1547 (57.6%)	276 (17.4%) 480 (30.3%) 829 (52.3%)	<0.001
Left ventricular ejection fraction (%)	50.0 [36.8–59.2]	52.0 [41.0–60.0]	57.0 [48.0–62.0]	58.0 [49.0–62.0]	<0.001

Data are median [25th; 75th percentiles] or number of patients (%). GFR = glomerular filtration rate.

**Table 2:** Ten-year clinical outcome.

Events	Estimated Glomerular Filtration Rate (mL/min/1.73 m^2^)				Hazard Ratio [95% Confidence Interval]		
	<30 (*n* = 198)	30 to <60 (*n* = 1104)	60 to <90 (*n* = 2684)	≥90 (*n* = 1585)	<30 vs. ≥90 mL/min/1.73 m^2^	30 to <60 vs. ≥90 mL/min/1.73 m^2^	60 to <90 vs. ≥90 mL/min/1.73 m^2^
All-cause death	155 (86.3)	602 (59.1)	775 (31.3)	220 (15.8)	13.35 [10.85–16.41]	5.16 [4.42–6.02]	2.15 [1.86–2.50]
Cardiac death	87 (49.0)	369 (37.0)	486 (20.0)	131 (9.7)	7.62 [5.78–10.05]	4.61 [3.78–5.63]	2.18 [1.80–2.65]
Noncardiac death	68 (37.3)	233 (22.1)	289 (11.3)	89 (6.1)	7.70 [5.61–10.57]	4.00 [3.13–5.10]	1.90 [1.50–2.42]
Myocardial infarction	17 (8.7)	84 (7.7)	155 (6.0)	78 (5.3)	1.81 [1.07–3.05]	1.55 [1.14–2.10]	1.16 [0.88–1.52]
Definite stent thrombosis	4 (2.1)	8 (0.7)	27 (1.0)	14 (0.9)	2.32 [0.76–7.04]	0.81 [0.34–1.93]	1.12 [0.59–2.13]
Target lesion revascularization	32 (16.9)	169 (15.7)	493 (19.0)	303 (20.7)	0.87 [0.60–1.27]	0.77 [0.64–0.93]	0.94 [0.82–1.09]
Target vessel revascularization	42 (21.8)	232 (21.4)	604 (23.1)	379 (25.1)	0.92 [0.67–1.28]	0.85 [0.72–1.00]	0.93 [0.82–1.06]
Nontarget vessel revascularization	43 (22.9)	287 (26.7)	767 (29.4)	462 (31.0)	0.76 [0.55–1.04]	0.87 [0.75–1.00]	0.96 [0.86–1.08]

Data are number of events with cumulative incidences calculated by the Kaplan–Meier method. For outcomes other than all-cause mortality, cumulative incidences were calculated after accounting for competing risk of death.

## Data Availability

The data that support the findings of this study are available from the corresponding author upon reasonable request.

## Supplementary Materials

The following supporting information can be downloaded at: https://www.mdpi.com/article/10.3390/jcm13226833/s1, Figure S1: Ten-year incidence of noncardiac mortality; Figure S2: Ten-year incidence of myocardial infarction; Figure S3: Ten-year incidence of definite stent thrombosis; Figure S4: Ten-year incidence of target-lesion revascularization; Figure S5: Ten-year incidence of target-vessel revascularization; Figure S6: Ten-year incidence of nontarget-vessel revascularization; Figure S7: Noncardiac mortality in subgroups of patients according to age, sex, diabetic status and clinical presentation; Figure S8: Myocardial infarction in subgroups of patients according to age, sex, diabetic status and clinical presentation; Figure S9: Stent thrombosis in subgroups of patients according to age, sex, diabetic status and clinical presentation; Figure S10: Unadjusted (left panel) and adjusted (right panel) association between glomerular filtration rate (GFR) and non-cardiac mortality; Figure S11: Putative mechanisms of the increased cardiometabolic risk and mortality in patients with impaired renal function; Table S1: Procedural data; Table S2: Drug therapy at hospital discharge; Table S3: Results of multivariable Cox proportional hazards model; Table S4: Association of estimated glomerular filtration rate categories with clinical outcomes after adjustment in the Cox proportional hazards model.
