# Supplementary material for: Impaired Kidney Function and 10-Year Outcome After Percutaneous Coronary Intervention—Interaction with Age, Sex, Diabetic Status and Clinical Presentation
Source: J Clin Med. 2024 Nov 13;13(22):6833. doi: 10.3390/jcm13226833 (PMC11594875; doi:10.3390/jcm13226833)
Supplement: Supplementary file 1 [file jcm-13-06833-s001.zip › jcm-3237014-supplementary.pdf]

Supplemental Material

Table S1. Procedural data

Table S2. Drug therapy at hospital discharge

Table S3. Results of multivariable Cox proportional hazards model

Table S4. Association of estimated glomerular filtration rate categories with clinical outcomes after adjustment in the Cox proportional hazards model

Figure S1. Ten-year incidence of noncardiac mortality

Figure S2. Ten-year incidence of myocardial infarction

Figure S3. Ten-year incidence of definite stent thrombosis

Figure S4. Ten-year incidence of target-lesion revascularization

Figure S5. Ten-year incidence of target-vessel revascularization

Figure S6. Ten-year incidence of nontarget-vessel revascularization

Figure S7. Noncardiac mortality in subgroups of patients according to age, sex, diabetic status and clinical presentation.

Figure S8. Myocardial infarction in subgroups of patients according to age, sex, diabetic status and clinical presentation.

Figure S9. Stent thrombosis in subgroups of patients according to age, sex, diabetic status and clinical presentation.

Figure S10. Unadjusted (left panel) and adjusted (right panel) association between glomerular filtration rate (GFR) and non-cardiac mortality

Figure S11. Putative mechanisms of the increased cardiometabolic risk and mortality in patients with impaired renal function

Table S1. Procedural data

Characteristic	Estimated glomerular filtration rate (ml/min/1.73 m^2)				P value
	<30<br>(n=274 lesions)	30 to <60<br>(n=1522 lesions)	60 to <90<br>(n=3727 lesions)	≥90<br>(n=2192 lesions)	
Vessel treated					0.004
LAD	114 (41.6%)	634 (41.7%)	1746 (46.8%)	961 (43.8%)	
LCX	64 (23.4%)	430 (28.3%)	917 (24.6%)	585 (26.7%)	
RCA	96 (35.0%)	458 (30.1%)	1064 (28.5%)	646 (29.5%)	
ACC/AHA complexity of lesions					0.025
A	5 (1.82%)	50 (3.3%)	164 (4.4%)	102 (4.6%)	
B1	45 (16.4%)	329 (21.6%)	864 (23.2%)	488 (22.3%)	
B2	153 (55.8%)	783 (51.4%)	1891 (50.7%)	1105 (50.4%)	
C	71 (25.9%)	360 (23.7%)	808 (21.7%)	497 (22.7%)	
Bifurcational lesions	591 (27.0%)	960 (25.8%)	396 (26.0%)	70 (25.5%)	0.775
Chronic occlusions	135 (6.2%)	196 (5.3%)	70 (4.6%)	20 (7.3%)	0.096
Reference diameter (mm)	2.79 [2.43-3.11]	2.79 [2.43-3.11]	2.77 [2.43-3.09]	2.76 [2.39-3.12]	0.786
Pre-procedural minimal lumen diameter (mm)	0.93 [0.64-1.26]	0.92 [0.63-1.24]	0.91 [0.63-1.21]	0.88 [0.66-1.21]	0.807
Baseline stenosis (%)	64.7 [55.6-75.4]	65.6 [55.5-76.1]	65.5 [56.5-75.5]	66.2 [56.8-75.5]	0.816
Balloon diameter (mm)	3.06 [2.68-3.47]	3.07 [2.67-3.49]	3.05 [2.65-3.45]	2.98 [2.60-3.45]	0.114
Maximal balloon pressure (atm)	16.0 [13.0-18.0]	16.0 [13.0-18.0]	16.0 [13.0-18.0]	15.2 [13.0-18.0]	0.936
Total stented length (mm)	24.0 [18.0-30.0]	23.0 [18.0-30.0]	24.0 [18.0-30.0]	24.0 [18.0-32.0]	0.067
Number of implanted stents	2.0 [1.0-2.0]	2.0 [1.0-2.0]	2.0 [1.0-2.0]	2.0 [1.0-2.0]	0.182
Post-procedural minimal lumen diameter (mm)	2.55 [2.24-2.89]	2.56 [2.23-2.88]	2.53 [2.22-2.86]	2.51 [2.20-2.82]	0.503
Residual stenosis (%)	11.2 [7.6-15.1]	10.9 [7.4-14.9]	10.8 [7.5-14.8]	10.8 [7.8-15.3]	0.656
Second generation DES	1940 (88.5%)	3336 (89.5%)	1354 (89.0%)	249 (90.9%)	0.504

Data are median with 25th-75th percentiles or counts (%); ACC=American College of Cardiology; AHA=American Heart Association; DES=drug-eluting stent; LAD=left anterior descending; LCX=left circumflex artery; RCA=right coronary artery

Table S2. Drug therapy at hospital discharge

Drug	Estimated glomerular filtration rate (ml/min/1.73 m^2)				P value
	<30 (n=198)	30 to <60 (n=1104)	60 to <90 (n=2684)	≥90 (n=1585)	
Aspirin	184/197 (93.4%)	1053/1098 (95.9%)	2637/2668 (98.8%)	1562/1575 (99.2%)	<0.001
Thienopyridines	172/197 (87.3%)	989/1096 (90.2%)	2516/2665 (94.4%)	1487/1574 (94.5%)	<0.001
Statins	162/194 (83.5%)	1007/1098 (91.7%)	2547/2668 (95.5%)	1520/1575 (96.5%)	<0.001
Angiotensin-converting enzyme inhibitors	112/198 (56.6%)	824/1104 (74.6%)	2094/2684 (78.0%)	1291/1585 (81.5%)	<0.001
Angiotensin II receptor blockers (AT1)	42/197 (21.3%)	209/1096 (19.1%)	462/2660 (17.4%)	219/1571 (13.9%)	0.001
Beta blockers	173/197 (87.8%)	1035/1098 (94.3%)	2501/2664 (93.9%)	1520/1575 (96.5%)	<0.001
Calcium channel blockers	65/196 (33.2%)	209/1097 (19.1%)	371/2662 (13.9%)	141/1579 (8.98%)	<0.001
Nitrates	7/196 (3.57%)	17/1094 (1.55%)	49/2661 (1.84%)	16/1569 (1.02%)	0.025
Diuretics	127/196 (64.8%)	776/1093 (71.0%)	1426/2660 (53.6%)	597/1567 (38.1%)	<0.001
Insulin	53/196 (27.0%)	159/1093 (14.5%)	177/2660 (6.65%)	109/1569 (6.95%)	<0.001
Oral antidiabetic drugs	25/197 (12.7%)	190/1093 (17.4%)	441/2662 (16.6%)	295/1569 (18.8%)	0.093
Warfarin	30/198 (15.2%)	224/1096 (20.4%)	312/2667 (11.7%)	74/1575 (4.70%)	<0.001

Data are numbers of patients (%)

Table S3. Results of multivariable Cox proportional hazards model applied for all-cause, cardiac and noncardiac mortality

Characteristic	All-cause mortality		Cardiac mortality		Noncardiac mortality	
	HR [95% CI]	P value	HR [95% CI]	P value	HR [95% CI]	P value
Estimated GFR (for 30 ml/min/1.73 m^2 decrement)	2.16 [1.84-2.54]	<0.001	2.15 [1.75-2.64]	<0.001	2.21 [1.71-2.86]	<0.001
Age (for 10-year increment)	2.05 [1.92-2.19]	<0.001	2.18 [2.00-2.37]	<0.001	1.87 [1.69-2.08]	<0.001
Women	0.95 [0.86-1.06]	0.389	1.04 [0.91-1.19]	0.556	0.82 [0.68-0.98]	0.030
Body mass index (for 5 kg/m^2 increment)	1.00 [0.94-1.06]	0.972	1.04 [0.97-1.12]	0.262	0.93 [0.85-1.03]	0.156
Diabetes mellitus	1.47 [1.33-1.63]	<0.001	1.57 [1.38-1.79]	<0.001	1.34 [1.14-1.58]	<0.001
Arterial hypertension	0.90 [0.81-1.00]	0.047	0.92 [0.80-1.05]	0.235	0.87 [0.73-1.02]	0.094
Current smoking	1.49 [1.28-1.72]	<0.001	1.66 [1.38-2.00]	<0.001	1.24 [0.97-1.58]	0.092
Hypercholesterolemia	0.90 [0.81-0.99]	0.038	0.94 [0.83-1.07]	0.348	0.84 [0.71-0.98]	0.029
Previous myocardial infarction	1.11 [1.00-1.23]	0.058	1.07 [0.93-1.22]	0.335	1.17 [0.99-1.39]	0.063
Previous coronary artery bypass surgery	1.04 [0.90-1.21]	0.566	1.02 [0.84-1.22]	0.876	1.10 [0.87-1.38]	0.440
Presentation with acute coronary syndrome	0.97 [0.88-1.07]	0.574	0.99 [0.87-1.12]	0.865	0.94 [0.80-1.10]	0.455
Multivessel disease	1.25 [1.07-1.47]	0.005	1.24 [1.02-1.52]	0.032	1.26 [0.97-1.63]	0.082
Type of coronary vessel	1.00 [0.90-1.12]	0.963	0.98 [0.85-1.13]	0.763	1.05 [0.88-1.26]	0.595
ACC/AHA complex lesions	1.00 [0.90-1.17]	0.976	1.01 [0.88-1.16]	0.888	0.99 [0.83-1.18]	0.916
Left ventricular ejection fraction (10% decrement)	1.32 [1.27-1.37]	<0.001	1.39 [1.32-1.46]	<0.001	1.22 [1.14-1.30]	<0.001

ACC/AHA=American College of Cardiology/American heart Association; CI=confidence interval; GFR=glomerular filtration rate; HR=hazard ratio

Table S4. Association of estimated glomerular filtration rate categories with clinical outcomes after adjustment in the Cox proportional hazards model

Outcome	Estimated glomerular filtration rate categories					
	<30 vs. ≥90 ml/min/1.73 m^2		30 to <60 vs. ≥90 ml/min/1.73 m^2		60 to <90 vs. ≥90 ml/min/1.73 m^2	
	HR [95% CI]	P value	HR [95% CI]	P value	HR [95% CI]	P value
All-cause death	4.34 [3.46 5.45]	<0.001	1.75 [1.46-2.09]	<0.001	1.10 [0.93-1.29]	0.256
Cardiac death	2.84 [1.75-4.59]	<0.001	1.80 [1.36-2.39]	<0.001	1.02 [0.82-1.28]	0.834
Noncardiac death	3.34 [1.99-5.62]	<0.001	1.79 [1.25-2.56]	<0.001	0.96 [0.72-1.27]	0.772
Myocardial infarction	1.95 [0.98-3.87]	0.056	1.53 [1.01-2.31]	0.045	1.17 [0.86-1.58]	0.319
Definite stent thrombosis	4.85 [1.17-20.06]	0.029	0.94 [0.37-2.35]	0.886	1.72 [0.86-3.44]	0.128
Target lesion revascularization	1.02 [0.65-1.60]	0.937	1.06 [0.81-1.38]	0.666	1.10 [0.94-1.30]	0.231
Target vessel revascularization	0.93 [0.63-1.38]	0.722	1.07 [0.85-1.34]	0.592	1.03 [0.89-1.20]	0.691
Nontarget vessel revascularization	0.81 [0.56-1.18]	0.281	1.01 [0.82-1.24]	0.936	1.10 [0.97-1.26]	0.148

CI=confidence interval; HR=hazard ratio

The group with preserved renal function (glomerular filtration rate ≥90 ml/min/1.73 m^2) served as reference.

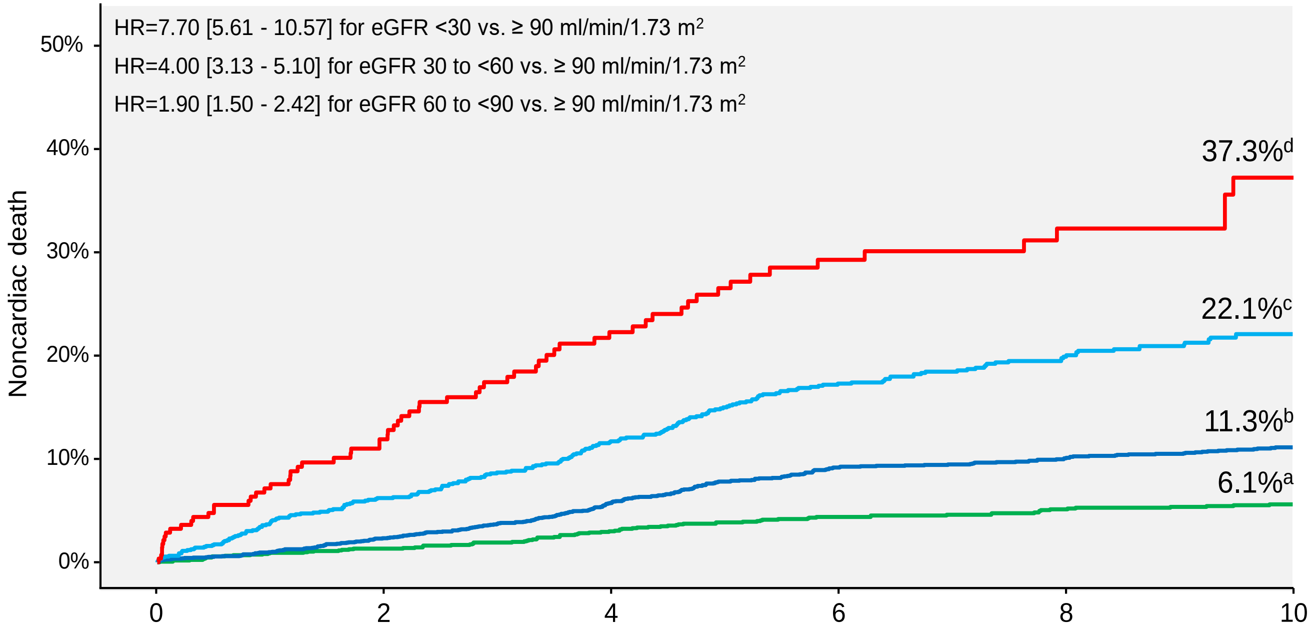

eGFR (ml/min/1.73 m²)	Numbers at risk					Years
	0	2	4	6	8	10
≥90[a]	1585	1494	1409	1235	1090	965
60 to <90[b]	2684	2508	2319	2020	1746	1438
30 to <60[c]	1104	942	811	633	464	333
<30[d]	198	122	80	49	28	17

Figure S1. Ten-year incidence of noncardiac mortality. eGFR = estimated glomerular filtration rate; HR=hazard ratio

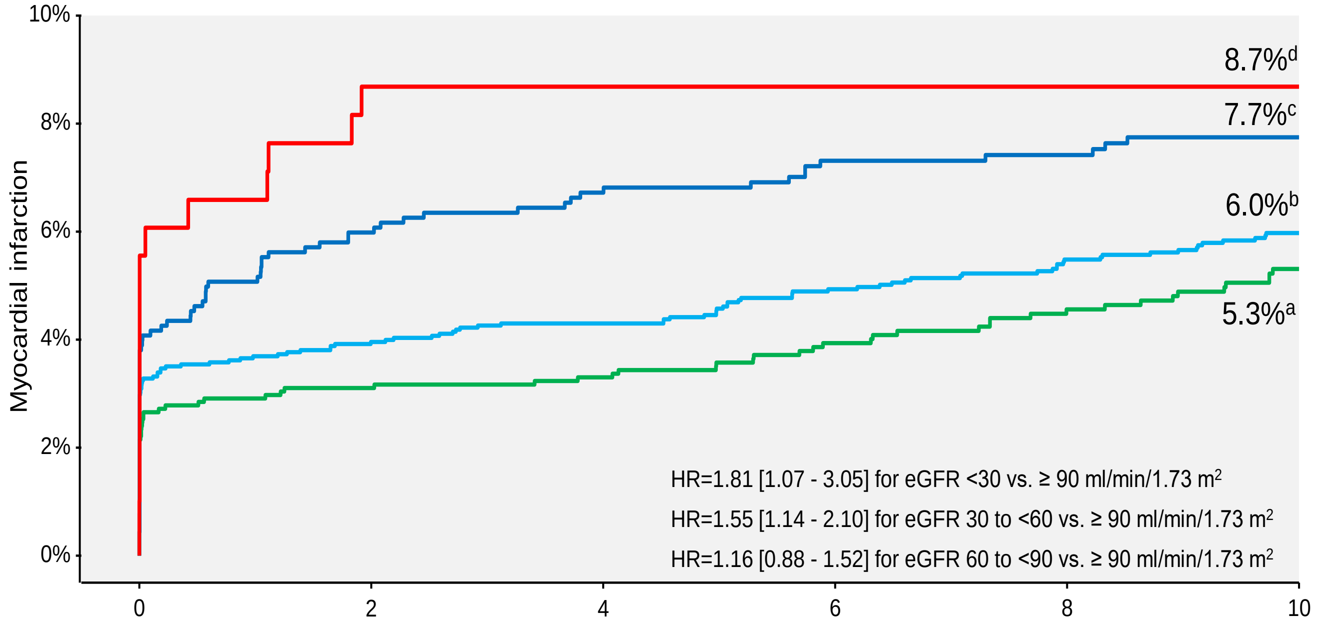

eGFR (ml/min/1.73 m^2)	Numbers at risk					Years
≥90[a]	1585	1454	1366	1186	1036	912
60 to <90[b]	2684	2416	2228	1927	1651	1356
30 to <60[c]	1104	904	767	594	438	312
<30[d]	198	112	76	48	27	16

Figure S2. Ten-year incidence of myocardial infarction. eGFR = estimated glomerular filtration rate; HR=hazard ratio

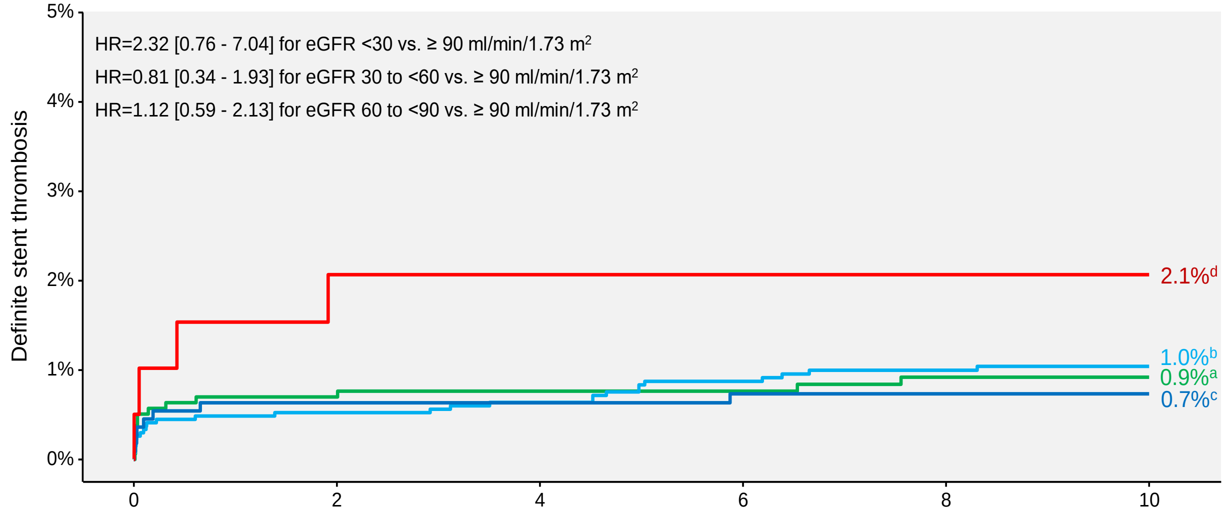

eGFR (ml/min/1.73 m²)	Numbers at risk					Years
≥90[a]	1585	1486	1400	1226	1079	958
60 to <90[b]	2684	2496	2305	2002	1727	1421
30 to <60[c]	1104	938	808	630	462	333
<30[d]	198	121	79	48	27	16

Figure S3. Ten-year incidence of definite stent thrombosis. eGFR = estimated glomerular filtration rate; HR=hazard ratio

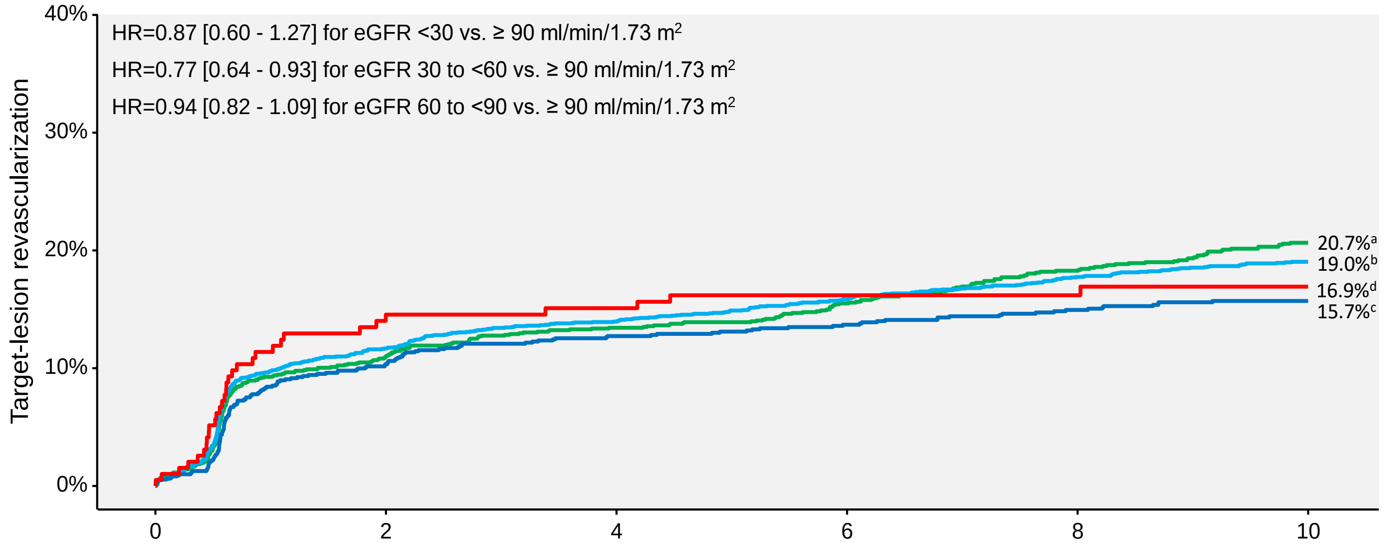

eGFR (ml/min/1.73 m²)	Numbers at risk					
≥90[a]	1585	1336	1222	1038	885	772
60 to <90[b]	2684	2213	1996	1698	1426	1155
30 to <60[c]	1104	835	696	532	373	266
<30[d]	198	101	65	38	21	14

Figure S4. Ten-year incidence of target-lesion revascularization. eGFR = estimated glomerular filtration rate; HR=hazard ratio

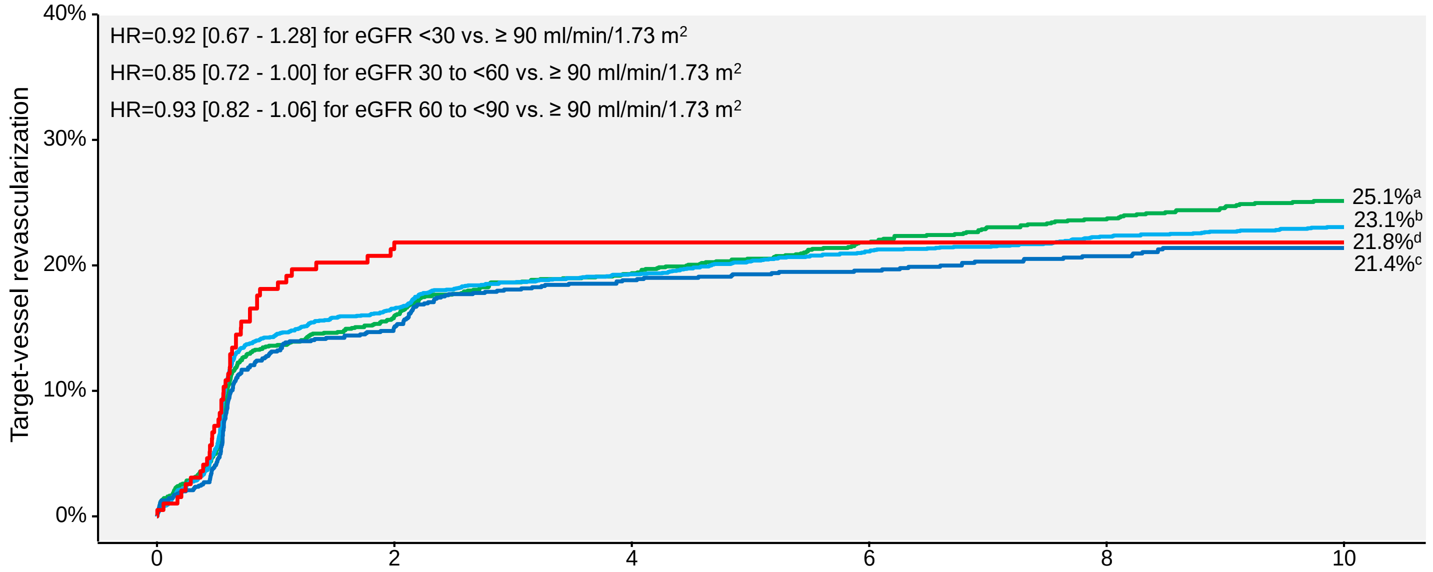

eGFR (ml/min/1.73 m^2)	Numbers at risk					
≥90[a]	1585	1264	1142	963	830	729
60 to <90[b]	2684	2091	1866	1580	1344	1101
30 to <60[c]	1104	787	642	493	345	247
<30[d]	198	91	59	34	20	14

Figure S5. Ten-year incidence of target-vessel revascularization. eGFR = estimated glomerular filtration rate; HR=hazard ratio

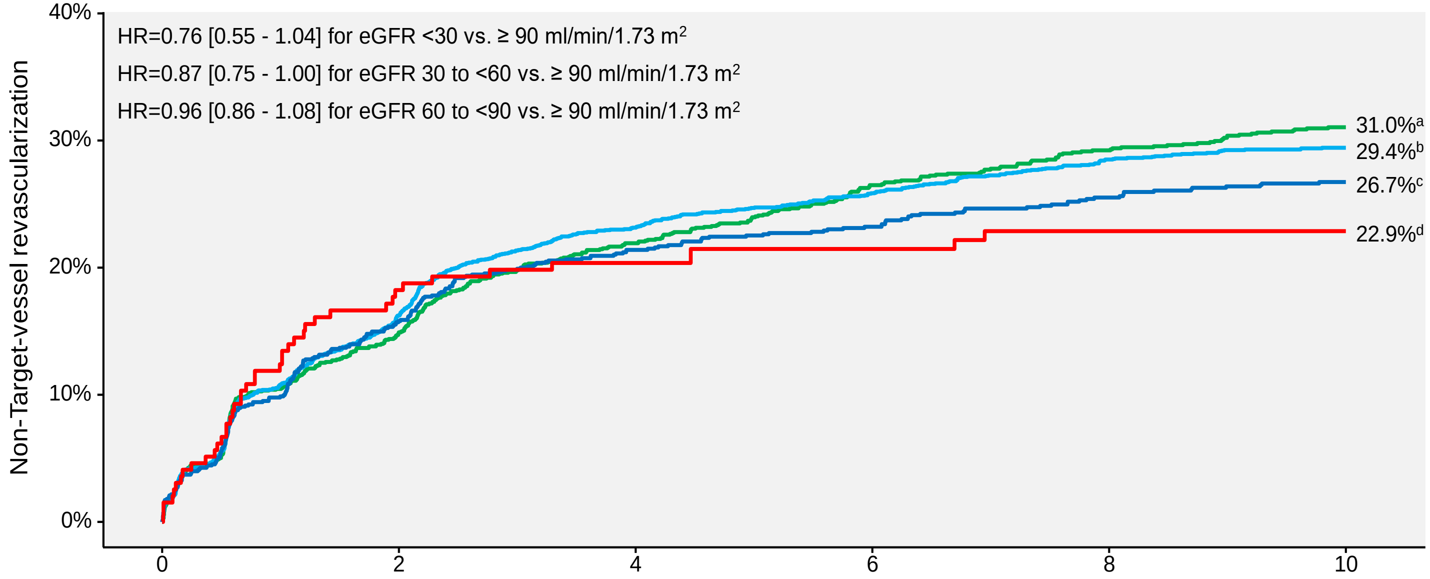

eGFR (ml/min/1.73 m²)	Numbers at risk					Years
≥90[a]	1585	1275	1099	897	753	649
60 to <90[b]	2684	2093	1764	1478	1204	982
30 to <60[c]	1104	785	625	478	326	229
<30[d]	198	91	58	36	19	13

Figure S6. Ten-year incidence of nontarget-vessel revascularization. eGFR = estimated glomerular filtration rate; HR=hazard ratio

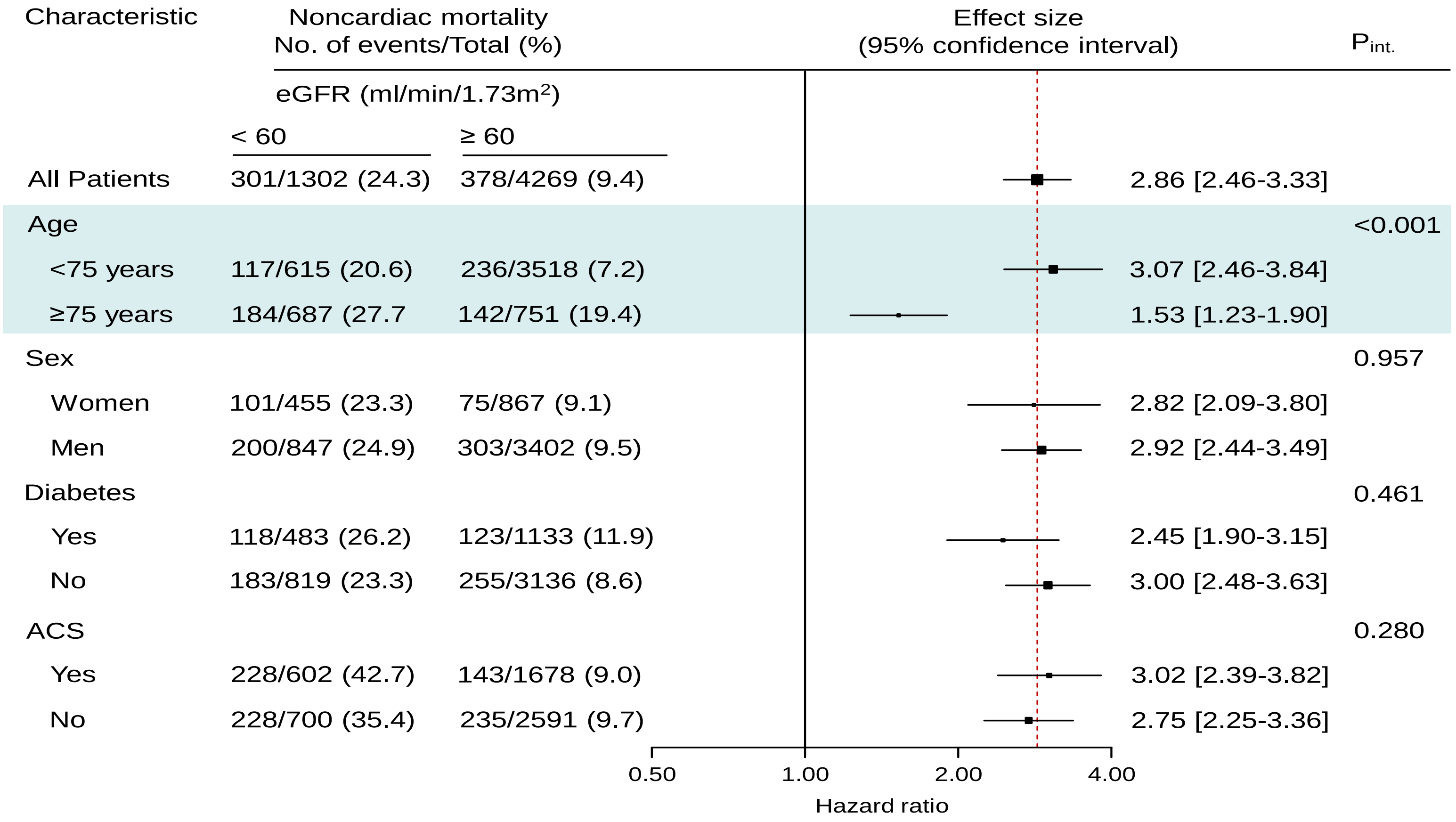

Figure S7. Noncardiac mortality in subgroups of patients according to age, sex, diabetic status and clinical presentation. ACS=acute coronary syndrome; P_{int} = P for interaction

Characteristic	Myocardial infarction No. of events/Total (%) eGFR (ml/min/1.73m²) < 60	≥ 60	Effect size (95% confidence interval)	$P_{int.}$
All Patients	101/1302 (24.3)	233/4269 (9.4)	1.44 [1.14-1.81]	
Age				0.022
<75 years	56/615 (9.3)	184/3518 (5.5)	1.78 [1.32-2.40]	
≥75 years	45/687 (6.7	49/751 (6.6)	1.00 [0.67-1.50]	
Sex				0.351
Women	39/455 (8.6)	65/867 (7.6)	1.15 [0.78-1.70]	
Men	62/847 (7.5)	168/3402 (5.2)	1.50 [1.12-2.00]	
Diabetes				0.489
Yes	45/483 (9.4)	70/1133 (6.6)	1.53 [1.05-2.21]	
No	56/819 (7.0)	163/3136 (5.4)	1.32 [0.98-1.79]	
ACS				0.361
Yes	54/602 (9.1)	99/1678 (6.2)	1.56 [1.12-2.16]	
No	47/700 (6.8)	134/2591 (5.4)	1.30 [0.93-1.81]	

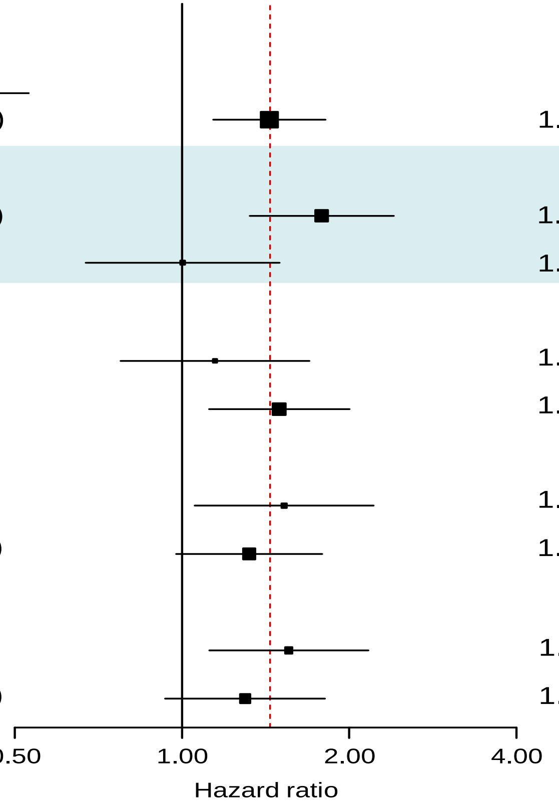

Figure S8. Myocardial infarction in subgroups of patients according to age, sex, diabetic status and clinical presentation. ACS=acute coronary syndrome; P_{int} = P for interaction

Characteristic	Stent thrombosis No. of events/Total (%) eGFR (ml/min/1.73m²) < 60	≥ 60	Effect size (95% confidence interval)	$P_{int.}$
All Patients	12/1302 (0.9)	41/4269 (1.0)	0.96 [0.50-1.82]	
Age				0.806
<75 years	9/615 (1.5)	38/3518 (1,1)	1.36 [0.66-2.81]	
≥75 years	3/687 (0.5)	3/751 (0.4)	1.11 [0.22-5.47]	
Sex				0.012
Women	8/455 (1.8)	5/867 (0.6)	3.06 [1.00-9.35]	
Men	4/847 (0.5)	36/3402 (1.1)	0.44 [0.16-1.24]	
Diabetes				0.400
Yes	8/483 (1.7)	17/1133 (1.6)	1.10 [0.47-2.55]	
No	4/819 (0.5)	24/3136 (0.8)	0.63 [0.22-1.83]	
ACS				0.486
Yes	7/602 (1.2)	17/1678 (1.0)	1.15 [0.48-2.78]	
No	5/700 (0.7)	24/2591 (1.0)	0.76 [0.29-2.00]	

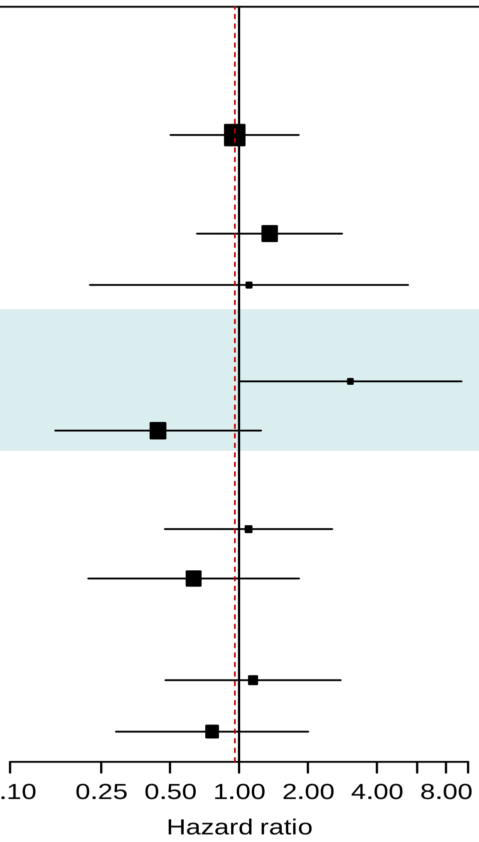

Figure S9. Stent thrombosis in subgroups of patients according to age, sex, diabetic status and clinical presentation. ACS=acute coronary syndrome; P_{int} = P for interaction

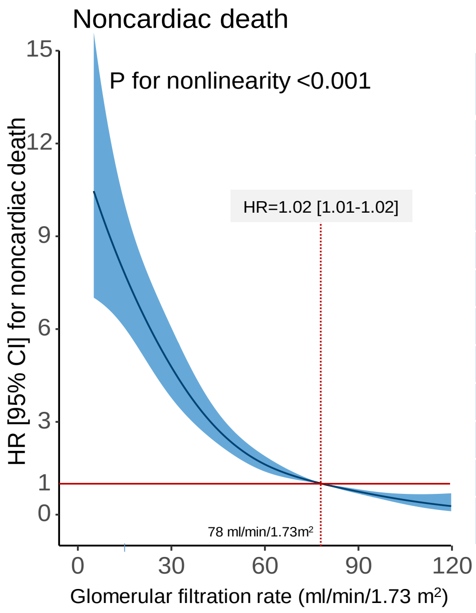

eGFR (ml/min/1.73 m²)	HR [95% CI]
15	7.82 [6.06 - 10.09]
30	4.80 [3.79 - 6.07]
40	3.28 [2.67 – 4.01]
50	2.28 [1.92 - 2.70]
60	1.63 [1.40 - 1.91]
70	1.22 [1.13 - 1.33]
80	0.96 [0.94 - 0.98]
90	0.75 [0.68 - 0.82]
100	0.55 [0.44 - 0.70]
110	0.40 [0.24 - 0.66]
120	0.28 [0.11 - 0.69]

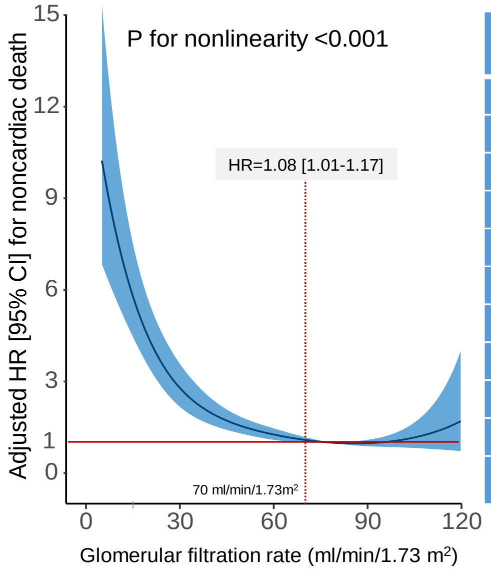

eGFR (ml/min/1.73 m²)	Adjusted HR [95% CI]
15	5.82 [4.48 - 7.56]
30	2.80 [2.18 - 3.60]
40	1.95 [1.57 - 2.43]
50	1.53 [1.28 - 1.82]
60	1.27 [1.09 - 1.47]
70	1.08 [1.00 - 1.17]
80	0.99 [0.97 - 1.01]
90	0.98 [0.88 - 1.09]
100	1.08 [0.84 - 1.37]
110	1.30 [0.79 - 2.12]
120	1.70 [0.72 - 4.00]

Figure S10. Unadjusted (left panel) and adjusted (right panel; the list of variables is shown in the methods) association between glomerular filtration rate (GFR) and non-cardiac mortality. The spline curves show the association between GFR values up to 120 ml/min/1.73 m^2 and noncardiac mortality at 10 years. The inserted tables on the right side of each graph show unadjusted (left panel) and adjusted (right panel) hazard ratios for noncardiac mortality for GFR values between 15 and 120 ml/min/1.73 m^2. For GFR values lower than 78 ml/min/1.73 m^2 (in unadjusted analysis) and 70 ml/min/1.73 m^2 (in adjusted analysis), the association between GFR and 10-year noncardiac mortality was significant. eGFR = estimated glomerular filtration rate; HR=hazard ratio

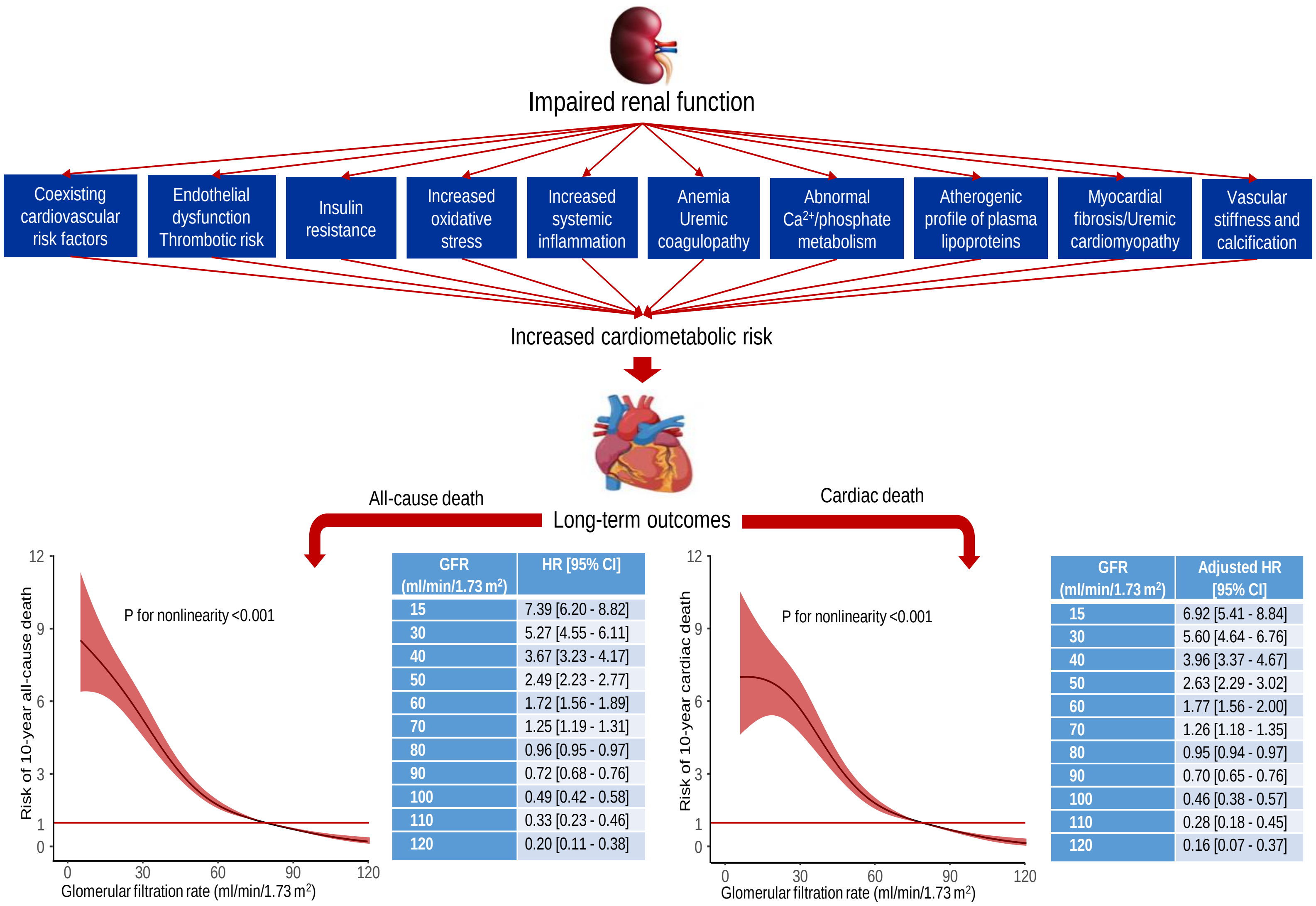

GFR (ml/min/1.73 m^2)	HR [95% CI]
15	7.39 [6.20 - 8.82]
30	5.27 [4.55 - 6.11]
40	3.67 [3.23 - 4.17]
50	2.49 [2.23 - 2.77]
60	1.72 [1.56 - 1.89]
70	1.25 [1.19 - 1.31]
80	0.96 [0.95 - 0.97]
90	0.72 [0.68 - 0.76]
100	0.49 [0.42 - 0.58]
110	0.33 [0.23 - 0.46]
120	0.20 [0.11 - 0.38]

GFR (ml/min/1.73 m^2)	Adjusted HR [95% CI]
15	6.92 [5.41 - 8.84]
30	5.60 [4.64 - 6.76]
40	3.96 [3.37 - 4.67]
50	2.63 [2.29 - 3.02]
60	1.77 [1.56 - 2.00]
70	1.26 [1.18 - 1.35]
80	0.95 [0.94 - 0.97]
90	0.70 [0.65 - 0.76]
100	0.46 [0.38 - 0.57]
110	0.28 [0.18 - 0.45]
120	0.16 [0.07 - 0.37]

Figure S11. Putative mechanisms of the increased cardiometabolic risk and mortality in patients with impaired renal function
